# The presence of neutralizing antibodies against omicron subvariants among a vaccinated cohort at one year after the first dose of vaccination in Malaysia

**DOI:** 10.1038/s41598-025-03798-8

**Published:** 2025-06-01

**Authors:** Eida Nurhadzira Muhammad, Zhuo Lin Chong, Mohd Amierul Fikri Mahmud, Filza Noor Asari, Rozainanee Mohd Zain, Masita Arip

**Affiliations:** 1 Center for Communicable Disease Epidemiology Research, Institute for Public Health, National Institute of Health, Shah Alam, Selangor Malaysia; 2 Infectious Disease Research Centre, Institute for Medical Research, National Institute of Health, Shah Alam, Selangor Malaysia; 3 Allergy and Immunology Research Centre, Institute for Medical Research, National Institute of Health, Shah Alam, Selangor Malaysia

**Keywords:** Neutralizing antibodies, Omicron variants, Omicron BA.1, BA.2, and BA.4/5, Vaccinated cohort, Malaysia, Disease prevention, Public health, Infectious-disease diagnostics, Vaccines

## Abstract

This study assesses the neutralizing antibodies response to Omicron subvariants and examines factors associated with seropositivity in a vaccinated Malaysian cohort. It is a prospective cohort study, conducted between June 2021 and October 2022. Descriptive and binary logistic regression analyses were performed on 1,117 adults aged 18 and above. Among the subvariants, seropositivity rates were: BA.2 (81.9%), BA.1 (68.4%), and BA.4/5 (64.2%). Non-Malaysians had significantly higher odds of testing positive for BA.2 compared to Malaysians (OR: 8.009; 95% CI: 1.273–50.402). Recipients of AstraZeneca (OR: 3.955; 95% CI: 2.414–6.482) and CanSino (OR: 1.980; 95% CI: 1.047–3.743) vaccines had higher odds of BA.2 seropositivity compared to Pfizer recipients. For BA.4/5, individuals aged 60 and above had greater odds of seropositivity (OR: 1.751; 95% CI: 1.029–2.979) compared to those aged 18–39. Chinese ethnicity was associated with lower odds of seropositivity than Malay ethnicity across BA.1 (OR: 0.508; 95% CI: 0.350–0.736), BA.2 (OR: 0.570; 95% CI: 0.377–0.861), and BA.4/5 (OR: 0.671; 95% CI: 0.467–0.963). This study highlights that completing primary vaccination and booster doses remains critical to reducing severe COVID-19 outcomes, underscoring the need for ongoing surveillance and targeted strategies for vulnerable demographic and socio-environmental groups.

## Introduction

 The Coronavirus Disease 2019 (COVID-19) pandemic was one of the most impactful public health emergencies of international concern. When the World Health Organization (WHO) declared the end of COVID-19 as a global emergency on May 5, 2023, there had been approximately 765,222,932 cases and nearly seven million deaths had been reported worldwide^1,2^. The causative agent of COVID-19 is Severe Acute Respiratory Syndrome Coronavirus 2 (SARS-CoV-2), which is a highly pathogenic virus from the Coronaviridae family that causes respiratory tract infections^3,4^. SARS-CoV-2 comprises four main structural proteins: envelope, membrane, nucleocapsid, and spike protein. Infection begins when the spike protein—specifically its receptor-binding domain (RBD)—binds to the angiotensin-converting enzyme 2 (ACE2) receptor on host cells, facilitating viral entry^5–8^. The virus continues to evolve through mutations, particularly in the spike protein, resulting in the emergence of new variants. A variant is classified when the virus acquires one or more significant genetic changes that affect key characteristics, such as transmissibility or immune evasion^6^. Over the course of the pandemic, several notable variants have emerged, including Alpha, Beta, Delta, and Omicron. More recently, Omicron subvariants such as BA.1, BA.2, BA.4, and BA.5 have gained prominence due to increased transmissibility and potential immune evasion. Additionally, newer variants, including XBB and BQ.1, continue to circulate, highlighting the ongoing evolution of SARS-CoV-2 and the need for continued surveillance and vaccination efforts.

As the COVID-19 pandemic unfolded, scientists and public health experts collaborated to develop effective solutions. The spike protein on the virus’s surface, which plays a crucial role in facilitating viral entry into human cells, became the primary target for vaccine development^5,9^. Leveraging data from previous coronavirus outbreaks and the early release of the SARS-CoV-2 genome on January 10, 2020, researchers were able to rapidly design vaccines. The full-length spike protein was selected as the vaccine antigen, forming the basis for most COVID-19 vaccines^10^. However, vaccine strategies based on the wild-type spike protein may not provide sustained protection against antigenically distinct variants, such as Omicron, due to the rapid evolution of SARS-CoV-2^11–13^. To evaluate post-vaccination protection, neutralizing antibodies levels can be measured. These antibodies are produced following both infection and vaccination^7,14^. They target the spike protein, preventing viral entry by altering its conformation and blocking its interaction with human cells^15^. Neutralizing antibodies function in tandem with binding antibodies, which recognize pathogens and alert the immune system to eliminate them. Although variants like Omicron harbor mutations that enable partial immune evasion, higher titers of neutralizing antibodies may still offer protection by reducing symptomatic infection and severe disease.

Neutralizing antibodies play a central role in vaccination. Vaccines stimulate the immune system through active immunization, prompting it to recognize and respond to the virus before actual exposure. Unlike passive immunity, which involves externally administered antibodies, active immunization engages the body’s adaptive immune response, offering long-term protection. In the case of SARS-CoV-2, the spike protein binds to the ACE2 receptor on host cells to facilitate viral entry. Vaccination-induced neutralizing antibodies block this interaction by binding to the spike protein, thereby preventing entry into the cells^16^. Nonetheless, the effectiveness of current vaccines is increasingly challenged by the accumulation of mutations in SARS-CoV-2 variants, particularly Omicron subvariants. These mutations may hinder the ability of neutralizing antibodies to bind efficiently to the spike protein, enabling immune escape. Consequently, the virus may still bind to ACE2 receptors, allowing entry and increasing the likelihood of breakthrough infections, even among vaccinated individuals^16^.

Malaysia experienced five epidemic waves of COVID-19^17^ with the first reported case of the wild-type virus in late January 2020, involving a Chinese tourist who had travelled from Singapore^18^. Subsequently, the Alpha and Beta variants were identified as the earliest variants of concern (VOCs) in December 2020, followed by the Delta variant, which spread widely between April and June 2021^17^. Meanwhile, the Omicron variant, which emerged in South Africa in November 2021, rapidly spread worldwide^19^. Beginning in December 2021, the Omicron BA.1 and BA.1.1 lineages began circulating in Malaysia, quickly displacing Delta as the dominant strain^18^. At the onset of the Omicron wave, three subvariants—BA.1, BA.2, and BA.3—were detected almost simultaneously. BA.1 and BA.2 share 21 common mutations but differ at 28 positions^20^. The Omicron BA.3 subvariant was identified alongside BA.1 and BA.2; however, BA.3 remained relatively rare in Malaysia and did not contribute significantly to case surges. Subsequently, Omicron BA.4 and BA.5 lineages were detected in South Africa, signaling their potential for global spread^20^. In Malaysia, BA.5 and its sub-lineages emerged in February 2022 and replaced the previously dominant BA.2 lineage^21^. These subvariants, which evolved from BA.2, share identical spike protein sequences but contain additional RBD mutations, such as L452R and F486 V. These mutations have been associated with reduced neutralization by antibodies, particularly among individuals vaccinated with mRNA vaccines^22^. The emergence of these highly transmissible variants significantly influenced global public health strategies, shaping vaccine policy, booster recommendations, and intervention measures. As these new variants exhibited greater immune evasion and potential for increased disease severity, health authorities continuously adapted their response strategies to mitigate transmission and safeguard public health.

In February 2021, Malaysia launched the COVID-19 National Immunization Program, beginning with the BNT162b2 vaccine produced by Pfizer. This initiative was subsequently expanded to include additional vaccines, such as CoronaVac from Sinovac and ChAdOx1 from AstraZeneca. The final vaccine received by Malaysia was Ad5-nCoV (CanSino). Beginning on 1 April 2022, individuals in Malaysia were required to comply with the national COVID-19 vaccination policy to be considered fully vaccinated^23^. As of November 9, 2024, Malaysia had achieved a significant milestone in its vaccination efforts: 84.4% of the total population—including adults, adolescents aged 12 to 17, and children aged five to eleven—had received their second dose of the COVID-19 vaccine. Additionally, approximately 50.1% of the population had received a booster dose, reflecting a strong national commitment to strengthening immunity against the virus^24^. As a result, by the end of 2024, Malaysia reported only 8,856 active cases, with no associated deaths^25^.

COVID-19 has now transitioned from a pandemic emergency to an endemic state worldwide. However, the possibility of new variant emergence remains, potentially leading to surges in cases and deaths^1^. Although the virus continues to circulate, its impact has significantly diminished compared to the peak of the pandemic in 2020 and 2021. The same trend has been observed in Malaysia. Accordingly, public health strategies and individual behaviours must evolve. Rather than mass vaccination campaigns, booster doses may be prioritized for high-risk groups, and continuous genomic surveillance of emerging variants remains essential for early outbreak detection. On an individual level, personal risk assessments—such as wearing masks in crowded settings and maintaining good hygiene practices—should become part of routine daily activities. Yet, a key question remains: are the vaccines administered during the pandemic still effective in preventing the spread of COVID-19, particularly the Omicron subvariants, in the current context or future outbreaks? This concern is especially relevant in Malaysia, where the primary vaccines and boosters used were developed based on the spike protein of the wild-type SARS-CoV-2 virus. Although studies have shown that these vaccines continue to offer substantial protection against severe disease, their ability to prevent infection has declined. To address this, bivalent mRNA booster vaccines—targeting both the ancestral strain and Omicron subvariants (BA.4/5)—have been developed. Research suggests that these bivalent boosters offer enhanced protection against severe COVID-19 outcomes compared to original monovalent vaccines. Furthermore, studies have shown that bivalent vaccines elicit higher levels of neutralizing antibodies against Omicron subvariants, indicating improved efficacy in preventing infection^26,27^. In this context, our study focused on a cohort of vaccinated individuals in Malaysia to evaluate whether the wild-type-based vaccination strategy continues to offer protection against Omicron subvariants one-year post-vaccination. Specifically, we aimed to assess the presence of neutralizing antibodies against Omicron subvariants (BA.1, BA.2, and BA.4/5) and identify factors associated with seropositivity in this vaccinated cohort. The findings are essential for evaluating Malaysia’s COVID-19 vaccination policy and offer valuable lessons for future pandemic preparedness and response.

## Methodology

### Study design and sampling of participants

This is a prospective cohort study conducted under the Post-vaccination COVID-19 Immunity and Disease Surveillance in Malaysia (IMSURE) project^28^ The cohort consisted of 2,513 adults aged 18 years and above who received COVID-19 vaccination under the National Immunization Program. For each of the four vaccines administered through the program, at least 600 recipients were selected non-probabilistically from various regions across Malaysia to ensure inclusivity. Participants were recruited at baseline before receiving their first dose of the vaccine and were followed up before their second dose and 14 days after that (or at 14 and 28 days after the single-dose vaccine). Follow-up continued every three months for up to 12 months from the first vaccination dose. During each follow-up, participants were interviewed, and a blood sample was collected. Recruitment took place between 17 June and 30 September 2021 (pre-Omicron period), and the study concluded on 4 October 2022 (post-Omicron). Retention efforts included contacting participants prior to each scheduled follow-up, including those who had missed previous sessions. Only participants who remained in the cohort at 12 months post-vaccination were included in the final analysis, resulting in a total of 1,117 participants (44.4%).

## Exposure variables

The exposure variables used in this study included sociodemographic characteristics (sex, age group, citizenship, and ethnicity), clinical characteristics (body mass index and comorbid status), and vaccination history (type of primary vaccination and booster status). All variables were collected through face-to-face interviews and document verification, except for the clinical characteristics. Body mass index was calculated using the standard formula based on the objectively measured weight and height of each participant. Comorbid status was self-reported and defined as having any one of the following conditions: diabetes mellitus, hypertension, asthma, chronic pulmonary disease, cancer, rheumatoid arthritis, systemic lupus erythematosus, Down syndrome, or diseases of the heart, liver, and kidneys, including kidney failure requiring haemodialysis. All exposure variables were collected at baseline before the first dose of vaccination, except for booster status, which was captured beginning at the six-month follow-up.

## Outcome variable

The primary outcome of this study was the presence of neutralizing antibodies against the SARS-CoV-2 Omicron subvariants BA.1, BA.2, and BA.4/5. Omicron BA.3 was excluded from the analysis due to its low prevalence in Malaysia. The GenScript cPass™ SARS-CoV-2 Neutralization Antibody Detection Kit (GenScript Biotech, United States) was used to perform the COVID-19 (Omicron) Surrogate Virus Neutralization Test (sVNT) on all samples. This assay complies with regulatory standards and has received approval or emergency use authorization from both the U.S. Food and Drug Administration (FDA) and the European Medicines Agency (EMA). It demonstrates a sensitivity of 99% and a specificity of 100%^29^.

The sVNT kit is a blocking ELISA detection technique that simulates the virus neutralization process. Its two primary components are the human ACE2 receptor protein (hACE2) and the horseradish peroxidase (HRP)-conjugated recombinant SARS-CoV-2 receptor-binding domain (HRP-RBD) fragment. Neutralizing antibodies against the SARS-CoV-2 RBD can prevent the interaction between HRP-RBD and hACE2. The assay was performed in accordance with the manufacturer’s instructions^30^. The procedure was generally divided into three main steps: the first step involved dilution of samples, positive controls, and negative controls; the second step included the preparation of positive/negative control samples and standard neutralization mixtures; and the third step involved the interaction of the neutralization reaction mixture with the ACE2-coated capture plate. The assay was conducted using a plate-based method. To prepare the sample and control dilution plate at a 1:10 ratio, an appropriate amount of sample dilution buffer was aliquoted into a reagent reservoir. Then, 72 µL of buffer and 8 µL of sample or control were transferred into each well using a multichannel micropipette. Next, the neutralization plate was prepared. A 1:1000 dilution of the HRP-RBD solution was aliquoted into the reagent reservoir, and 60 µL of the diluted HRP-RBD was pipetted into all wells. This was followed by adding 60 µL of serially diluted standard solution into each respective well. Prior to pipetting, each tube of the standard solution was vortexed and lightly centrifuged to ensure complete mixing. Then, 60 µL of the 1:10 diluted samples and controls were transferred from the dilution plate into the neutralization plate. The mixtures were gently resuspended using a multichannel pipette to ensure even mixing. The neutralization reaction mixture was then incubated at 37 °C for 30 min. Following incubation, 100 µL of the HRP-RBD neutralization mixture was transferred from the neutralization plate to the ACE2-coated capture plate and incubated at 37 °C for 15 min. The plate was then washed four times, and residual liquid was removed completely. Finally, the substrate reaction and absorbance measurement were carried out. The results can be interpreted by comparing the inhibition rate to the positive and negative cutoffs for SARS-CoV-2 neutralizing antibodies detection.

$$\%{\rm Inhibition}=\left[1-\frac{\rm Optical\: Density\: (OD)\: value\: of\: sample}{\rm OD\: value\: of\: negative\: control}\: \right] \rm X\: 100\%$$

Based on the formula above, an inhibition percentage of 30% or higher indicates the presence of detectable SARS-CoV-2 neutralizing antibodies. Conversely, an inhibition percentage below 30% suggests that no detectable neutralizing antibodies are present^30^.

## Data management

At the end of each data collection day, all information gathered using mobile tablets was securely synchronized via the internet with encrypted servers. The core dataset was then integrated with traceable laboratory results. Access to the password-protected merged dataset was restricted to data managers and investigators only. The data obtained is considered strategic and valuable for Malaysia, particularly in the prevention and control of infectious diseases with pandemic potential. Consequently, it has been securely stored indefinitely for future analyses and research.

### Data analysis

Descriptive analysis was conducted to examine the distribution of participants based on sociodemographic characteristics and seropositivity to Omicron subvariants (BA.1, BA.2, and BA.4/5). Chi-square analysis was used to assess significant differences between Omicron subvariants and various variables. To evaluate the strength of associations between Omicron subvariant seropositivity (dependent variable) and categorical independent variables (sex, age group, ethnicity, citizenship, body mass index, comorbid status, type of primary vaccination, and booster status), binary logistic regression was applied. Only odds ratios (ORs) with a p-value less than or equal to 0.25 were included in the multivariable model for adjusted ORs. A p-value of ≤ 0.05 was considered statistically significant.

### Result

A total of 1,117 participants were included in this study after successfully completing the one-year post-vaccination follow-up. The mean age of participants was 38.62 ± 14.06 years, with ages ranging from 17 to 84 years. The participants’ sociodemographic distribution and positivity rates for Omicron subvariants are summarized in Table 1. Among the Omicron subvariants, BA.2 exhibited the highest positivity rate (81.9%), followed by BA.1 (68.4%) and BA.4/5 (64.2%). Chi-square analysis revealed significant associations between subvariant positivity and several demographic and clinical factors. Omicron BA.1 was significantly associated with citizenship (*p* = 0.002), ethnicity (*p* < 0.001), body mass index (BMI) (*p* = 0.032), and booster status (*p* = 0.042). Omicron BA.2 showed significant differences based on citizenship (*p* = 0.050) and ethnicity (*p* = 0.004). Omicron BA.4/5 was significantly associated with age group (*p* = 0.015) and comorbidity status (*p* = 0.019). All three Omicron subvariants (BA.1, BA.2, and BA.4/5) were significantly associated with the type of primary COVID-19 vaccination, with p-values of 0.003, < 0.001, and 0.003, respectively.

In the final logistic regression model, after adjusting for confounding factors, only Chinese and Bumiputera Sabah ethnicities showed a significant association with seropositivity for Omicron BA.1 (*p* < 0.001). The odds of seropositivity for Omicron BA.1 among individuals of Chinese and Bumiputera Sabah ethnicities were 0.508 (95% CI: 0.350–0.736) and 0.207 (95% CI: 0.094–0.456), respectively, compared to those of Malay ethnicity (Table 2). Additionally, several factors including Chinese ethnicity, non-Malaysian citizenship, and vaccination with AstraZeneca or CanSino were significantly associated with seropositivity for Omicron BA.2 (*p* < 0.05). Specifically, Chinese ethnicity was associated with an odds ratio (OR) of 0.570 (95% CI: 0.377–0.861) for seropositivity to Omicron BA.2 compared to Malay ethnicity, while non-Malaysian individuals had significantly higher odds (OR: 8.009; 95% CI: 1.273–50.402) of testing positive compared to Malaysian citizens. Furthermore, individuals vaccinated with AstraZeneca and CanSino had a 3.955-fold (95% CI: 2.414–6.482) and 1.980-fold (95% CI: 1.047–3.743) increased likelihood of testing positive for Omicron BA.2, respectively, compared to those who received the Pfizer vaccine. For Omicron BA.4/5, individuals aged 60 years and above had significantly higher odds of seropositivity (OR: 1.751; 95% CI: 1.029–2.979) compared to those aged 18–39 years. Consistent with the findings for Omicron BA.1 and BA.2, Chinese ethnicity also remained significantly associated with Omicron BA.4/5 positivity (*p* = 0.031), with an OR of 0.671 (95% CI: 0.467–0.963).

**Table 1:** Participants’ sociodemographic distribution and positivity of Omicron variants (*N* = 1,117).

Variables	Total, *n*	Positivity of Omicron variants, *n* (%)					
		BA 1	*p*-value	BA 2	*p*-value	BA 4/5	*p*-value
Total	1,117	764 (68.4)	-	915 (81.9)	-	717 (64.2)	-
Sex							
*Male*	548	361 (65.9)	0.082	447 (81.6)	0.816	359 (65.5)	0.382
*Female*	569	403 (70.8)		468 (82.2)		358 (62.9)	
Age group							
*18–39 years old*	644	434 (67.4)	0.700	526 (81.7)	0.902	398 (61.8)	0.015*
*40–59 years old*	364	254 (69.8)		298 (81.9)		236 (64.8)	
*≥ 60 Years old*	109	76 (69.7)		91 (83.5)		83 (76.1)	
Citizenship							
*Malaysian*	1022	713 (69.8)	0.002*	830 (81.2)	0.050*	652 (63.8)	0.434
*Non-Malaysian*	95	51 (53.7)		85 (89.5)		65 (68.4)	
Ethnicity							
*Malay*	713	525 (73.6)	< 0.001*	593 (83.2)	0.004*	463 (64.9)	0.079
*Chinese*	170	101 (59.4)		124 (72.9)		93 (54.7)	
*Indian*	71	49 (69.0)		55 (77.5)		50 (70.4)	
*Bumiputera Sabah*	39	13 (33.3)		37 (94.9)		25 (64.1)	
*Bumiputera Sarawak*	24	22 (91.7)		19 (79.2)		18 (75.0)	
*Others*	100	54 (54.0)		87 (87.0)		68 (68.0)	
Body Mass Index (BMI)							
*Underweight*	68	38 (55.9)	0.032*	53 (77.9)	0.452	38 (55.9)	0.11
*Normal*	255	168 (65.9)		212 (83.1)		152 (59.6)	
*Overweight*	337	228 (67.7)		269 (79.8)		223 (66.2)	
*Obese*	457	330 (72.2)		381 (83.4)		304 (66.5)	
Comorbid status							
*No*	663	449 (67.7)	0.600	531 (80.1)	0.058	407 (61.4)	0.019*
*Yes*	454	315 (69.4)		384 (84.6)		310 (68.3)	
Type of primary series Covid-19 vaccine received							
*Pfizer*	277	207 (74.7)	0.003*	203 (73.3)	< 0.001*	189 (68.2)	0.003*
*Sinovac*	307	214 (69.7)		281 (91.5)		208 (67.8)	
*AstraZeneca*	316	215 (68.0)		239 (75.6)		176 (55.7)	
*CanSino*	217	128 (59.0)		192 (88.5)		144 (66.4)	
Booster status							
*No*	392	252 (64.3)	0.042*	322 (82.1)	0.631	244 (62.2)	0.211
*Yes*	721	508 (70.6)		589 (81.8)		469 (65.2)	

*Significant association (p < 0.05)

**Table 2:** Factors associated with neutralizing antibodies positivity against Omicron subvariants.

Variables	Omicron BA.1				Omicron BA.2					Omicron BA.4/5				
	Crude OR (95% CI)	*p* value	Adjusted OR (95% CI)	*p* value		Crude OR (95% CI)	*p* value	Adjusted OR (95% CI)	*p* value		Crude OR (95% CI)	*p* value	Adjusted OR (95% CI)	*p* value
Sex														
*Male*	*Reference*					*Reference*					*Reference*			
*Female*	1.258 (0.977, 1.619)	0.075	1.219 (0.935, 1.588)	0.143		1.047 (0.772, 1.420)	0.768	-			0.893 (0.699, 1.141)	0.366	-	
Age category														
*18–39 years*	*Reference*					*Reference*					*Reference*			
*40–59 years*	1.117 (0.846, 1.475)	0.434	-			1.013 (0.726, 1.413)	0.94	-			1.140 (0.872, 1.489)	0.338	-	
*≥ 60 Years old*	1.114 (0.717, 1.731)	0.630	-			1.134 (0.659, 1.953)	0.65	-			1.973 (1.235, 3.152)	0.004**	1.751 (1.029, 2.979)	0.039**
Ethnicity														
*Malay*	*Reference*					*Reference*					*Reference*			
*Chinese*	0.524 (0.370, 0.743)	< 0.001**	0.508 (0.350, 0.736)	< 0.001**		0.545 (0.369, 0.807)	0.002**	0.570 (0.377, 0.861)	0.008**		0.652 (0.465, 0.915)	0.013**	0.671 (0.467, 0.963)	0.031**
*Indian*	0.798 (0.470, 1.355)	0.403	-			0.696 (0.385, 1.255)	0.228	0.754 (0.408, 1.394)	0.368		1.286 (0.755, 2.189)	0.355	-	
*Bumiputera Sabah*	0.179 (0.090, 0.356)	< 0.001**	0.207 (0.094, 0.456)	< 0.001**		3.744 (0.890, 15.743)	0.072	3.021 (0.663, 13.722)	0.153		0.964 (0.492, 1.888)	0.915	-	
*Bumiputera Sarawak*	3.939 (0.917, 16.912)	0.065	3.565 (0.813, 15.631)	0.092		0.769 (0.282, 2.100)	0.608	-			1.620 (0.635, 4.133)	0.313	-	
*Others*	0.420 (0.274, 0.644)	< 0.001**	0.597 (0.126, 2.842)	0.517		1.354 (0.732, 2.505)	0.334	-			1.147 (0.733, 1.795)	0.547	-	
Citizenship														
*Malaysian*	*Reference*					*Reference*					*Reference*			
*Non-Malaysian*	0.502 (0.328, 0.768)	0.001**	0.699 (0.137, 3.557)	0.666		4.323 (1.002, 3.857)	0.049**	8.009 (1.273, 50.402)	0.027**		1.230 (0.783, 1.930)	0.369	-	
Body Mass Index (BMI)														
*Normal*	*Reference*					*Reference*					*Reference*			
*Underweight*	0.683 (0.406, 1.149)	0.151	0.721 (0.414, 1.257)	0.249		0.815 (0.436, 1.521)	0.520	-			0.799 (0.476, 1.342)	0.397	-	
*Overweight*	1.459 (1.070, 1.990)	0.017**	1.349 (0.979, 1.860)	0.067		1.145 (0.788, 1.664)	0.478	-			1.370 (1.015, 1.849)	0.040**	1.479 (0.854, 2.561)	0.163
*Obese*	1.297 (0.937, 1.796)	0.117	1.087 (0.769, 1.536)	0.636		1.062 (0.718, 1.571)	0.762	-			1.174 (0.857, 1.606)	0.317	-	
Comorbid status														
*No*	*Reference*					*Reference*					*Reference*			
*Yes*	1.080 (0.835, 1.397)	0.558	-			1.364 (0.992, 1.874)	0.056	1.195 (0.850, 1.680)	0.305		1.354 (1.052, 1.742)	0.018**	1.135 (0.853, 1.511)	0.385
Type of primary vaccination														
*Pfizer*	*Reference*					*Reference*					*Reference*			
*AstraZeneca*	0.778 (0.541, 1.120)	0.177	0.818 (0.554, 1.207)	0.312		3.940 (2.433, 6.379)	< 0.001**	3.955 (2.414, 6.482)	< 0.001**		0.978 (0.691, 1.386)	0.901	-	
*Sinovac*	0.720 (0.502, 1.031)	0.073	0.816 (0.561, 1.188)	0.289		1.131 (0.782, 1.638)	0.513	-			0.585 (0.418, 0.820)	0.002**	0.748 (0.517, 1.080)	0.121
*CanSino*	0.486 (0.332, 0.713)	< 0.001**	1.001 (0.575, 1.744)	0.997		2.800 (1.707, 4.590)	< 0.001**	1.980 (1.047, 3.743)	0.036**		0.918 (0.629, 1.341)	0.660	-	
Booster status														
*No*	*Reference*					*Reference*					*Reference*			
*Yes*	1.335 (1.028, 1.734)	0.030**	1.260 (0.904, 1.756)	0.173		0.977 (0.709, 1.345)	0.885	-			1.138 (0.882, 1.469)	0.319	-	

**Indicates significant differences; p < 0.05; OR-Odd Ratio

## Discussion

Prior research revealed that vaccinated individuals’ serum samples neutralized the Omicron variants to a significantly lower extent than the other variants examined, such as Alpha, Beta, or Delta^13,31,32^. This phenomenon arises from the Omicron variant’s ability to resist the immune response triggered by previous infections or vaccinations based on the original SARS-CoV-2 strain^33,34^. Omicron BA.2, which was predominantly detected in several nations like Denmark, Nepal, and the Philippines, gradually replaced Omicron BA.1, the most prevalent strain in the world^34–36^. In contrast, the emergence of Omicron BA.3 was marked by very limited transmissibility, with only a few cases worldwide. Finally, Omicron BA.4/5 was discovered and subsequently replaced Omicron BA.2 as the dominant strain^35^. This study compared positivity rates among the Omicron subvariants (BA.1, BA.2, and BA.4/5) and found that Omicron BA.2 had a higher positivity rate than BA.1 and BA.4/5. Notably, less than two weeks after the Omicron variant was initially discovered, the BA.2 lineage emerged among the Omicron lineages. It expanded more quickly than the BA.1 lineage before becoming the dominant lineage^35,36^. A study conducted by Jiahui C et al. revealed that BA.2 is approximately 1.5 times more contagious than BA.1 and 20 times more contagious than the original SARS-CoV-2^37^. Meanwhile, Omicron BA.4/5 demonstrated the lowest positivity rate compared to other Omicron subvariants, specifically BA.1 and BA.2. The spike sequences of Omicron BA.4/5 closely resembled those of BA.2, but their spike protein had alterations that increased resistance to neutralizing antibodies^38^. Specific spike protein mutations in Omicron BA.4/5 and BA.2, such as the L452R mutation, enhanced the spike protein’s affinity for the ACE2 receptor, facilitating viral entry into host cells. Additionally, this mutation reduced the neutralizing capacity of certain antibodies, contributing to immune escape^39^. As a result, lower inhibition percentages were observed, as these changes diminished the effectiveness of antibodies generated from earlier infections or vaccinations. Furthermore, neutralization titers against Omicron BA.4/5 were substantially lower than those against BA.1 and BA.2, as reported by Aekkachai T. et al. and other studies^13,22^. Lower levels of neutralizing antibodies suggest that vaccines may provide reduced protection against infection with Omicron variants compared to earlier strains. Studies have also indicated increased odds of hospitalization and death beyond three months after the third vaccine dose during the Omicron period^40^. Moreover, a decline in neutralization can contribute to an increase in breakthrough infections among vaccinated individuals and a potential rise in reinfections for individuals who have recovered from previous COVID-19 cases. Enhancing vaccine formulations could improve the efficacy of the COVID-19 vaccine against variants like Omicron BA.4/5 and other emerging variants. Specifically, developing vaccines that target key mutations in circulating variants like BA.4/5 could enhance neutralization. Malaysia did not administer a booster with an improved formulation. However, our results suggest that the population likely retains a certain level of protection against Omicron subvariants. In retrospect, this provides a valuable lesson for Malaysia. However, continuous genomic surveillance and proactive public health measures are essential to monitor emerging variants and ensure timely interventions to mitigate the risk of breakthrough infections and reinfections.

In contrast to Malaysia, where multiple Omicron subvariants have emerged and demonstrated varying seropositivity rates among vaccinated individuals, some neighbouring regions have experienced a more limited diversity of circulating variants. For instance, during the first COVID-19 wave in Bali, Indonesia, whole-genome sequencing revealed that none of the detected SARS-CoV-2 strains were classified as variants of concern. Most isolates belonged to the B.1.466.2 lineage and carried the D614G spike mutation, with no evidence of major immune-evading mutations, such as those found in Omicron subvariants^42^. This limited genomic diversity is likely attributable to strict public health interventions, including travel restrictions and mobility controls implemented early in the pandemic. Conversely, a study from Uttar Pradesh, India, comparing genomic data from the first and second waves, documented a substantial increase in mutation density during the second wave, especially in the spike protein region, coinciding with the emergence of the Delta variant and its subclades^43^. The dynamic and regionally distinct evolution of SARS-CoV-2 highlights how differences in public health responses, mobility patterns, and vaccination coverage shape viral transmission and variant emergence. These findings reinforce the importance of sustained genomic surveillance and responsive public health strategies tailored to local epidemiological contexts.

In this study, we determined the factors associated with the positivity rates of Omicron BA.1, BA.2, and BA.4/5. We found a lower significant association between Chinese ethnicity in Malaysia and the positivity rate of all Omicron subvariants (BA.1, BA.2, and BA.4/5). Although specific studies detailing this association are limited, several factors may contribute to the observed disparity. Chinese individuals might exhibit different social behaviours and cultural practices, such as higher rates of mask-wearing, stricter adherence to social distancing, and reduced participation in crowded events, all of which could lead to lower exposure rates. Socioeconomic factors may also play a role, as they are more likely to live in less crowded housing and work in sectors that allow for remote work. In addition, their access to healthcare services is generally more efficient. Higher or earlier vaccination uptake among the Chinese population may have further enhanced their protection, resulting in lower infection rates. Apart from Chinese ethnicity, the Bumiputera Sabah ethnicity was also significantly associated with Omicron positivity, but only for the BA.1 subvariant. Sabah is recognized as one of the Malaysian states most significantly impacted by SARS-CoV-2, experiencing high transmission rates of the Omicron variant. A previous study conducted in Sabah identified BA.1 as the most prevalent lineage, with many sequenced cases classified as BA.1^44^. The BA.1 and BA.1.1 lineages emerged as dominant variants in the eastern region of Malaysia, with the initial detection of the Omicron variant B.1.1 reported in Sabah in February 2022^18^, coinciding with the data collection period for this study. However, Sabah’s rural and less densely populated areas may have experienced reduced exposure to the virus, leading to lower infection rates.

Our findings revealed a significant association between Omicron BA.2 positivity and non-Malaysian individuals. Omicron BA.2 differs greatly from BA.1 in its virological traits due to its antigenically distinct and significantly different genomic sequence^36^. At least five BA.2 genetic subgroups have emerged in various geographic locations, indicating ongoing antigenic drift within this subvariant^45^. Following its initial detection in India in May 2022, BA.2.75 (a sublineage of Omicron BA.2) was increasingly detected in several regions of the world, with different sublineages of BA.2.75 achieving high prevalence in different countries. BA.2 surpassed BA.1 only in isolated circumstances, reaching high prevalence in Denmark, India, and Nepal before spreading to other Asian countries, such as Singapore, the Philippines, and China^34,45–47^. This could be one of the reasons for the higher prevalence of Omicron BA.2 among non-Malaysians, as Malaysia has become a major destination for migrant workers, especially from Indonesia, Bangladesh, Nepal, and India. These workers are often employed in manufacturing, construction, plantation, and domestic work due to their willingness to work long hours^48^. A considerable proportion of migrants in Malaysia, particularly migrant workers, reside in communal settings such as hostels, stacked containers, and accommodation blocks, which often lead to overcrowded living conditions. These environments facilitate the rapid spread of the virus, increasing community transmission during the study period. As a result, outbreaks or clusters may disproportionately impact specific populations, highlighting the vulnerabilities associated with densely populated living arrangements. Beyond the migrant workforce, Malaysia is also a major tourist destination. The Malaysian government announced that beginning on April 1, 2022, all travellers would be allowed to enter the country^49^. Cultural and behavioural differences, including varying practices regarding mask-wearing, social distancing, and adherence to public health guidelines, could also influence transmission rates. Meanwhile, differences in age, underlying health conditions, and overall health status between non-Malaysians and Malaysians may also contribute to varying infection rates.

According to the type of vaccination received, AstraZeneca and CanSino were significantly associated with higher Omicron BA.2 positivity rates compared to Pfizer. Through our literature search, we did not find any studies directly examining the association between AstraZeneca or CanSino and seropositivity to Omicron BA.2. However, this finding may be influenced by vaccine characteristics, variant evolution, and immune response. Different vaccines may induce distinct antibody profiles and varying immunological responses in the same individual. Furthermore, the immune response to vaccination can differ between individuals due to factors such as ethnicity, gender, age, and underlying medical conditions^38^. Compared with mRNA vaccines, adenovirus-vector vaccines—such as AstraZeneca and CanSino—exhibit multiple levels of complexity that can trigger diverse immune responses. These factors include the gene design for S protein (spike) expression, genetic modifications made to the vector, and the type of adenovirus vector used^50^. The CanSino vaccine, being a single-dose regimen, generally induces a lower initial immune response compared to two-dose vaccines. This reduced immune response may contribute to increased susceptibility to breakthrough infections with immune-evasive variants like BA.2, particularly in the absence of booster doses. Individuals who received the CanSino vaccine without subsequent boosters may have faced a higher risk of infection. BA.2’s strong immune escape characteristics, combined with the lower antibody response from a single-dose vaccine, may have contributed to an elevated positivity rate in individuals vaccinated with CanSino.

Based on age group analysis, our study found a significant association between Omicron BA.4/5 positivity and participants aged 60 years and older. Older individuals are generally more vulnerable to infection due to a combination of weakened immune systems, delayed or insufficient booster coverage, and increased exposure to the virus in healthcare and communal settings. Compared to BA.1 and BA.2, the Omicron BA.4/5 subvariants harbor additional mutations in their spike protein, particularly in the receptor-binding domain^51,52^. These mutations enhance immune evasion, reducing protection conferred by prior infections or vaccinations and thereby increasing susceptibility among older individuals, who may exhibit weaker immune responses. Older adults typically show reduced levels of neutralizing antibodies due to immunosenescence—a natural decline in immune function with age. This decline impairs their ability to mount a robust response to emerging variants such as BA.4/5, even if they have been vaccinated or previously infected with BA.1 or BA.2. A prior study indicated that the BA.4/5 subvariants exhibit greater pathogenicity than BA.1 and BA.2 and demonstrate significant resistance to immunity elicited by these earlier variants^52^. Although vaccines provide protection against severe disease, BA.4/5 can partially evade neutralizing antibodies generated by prior vaccination—especially in the absence of booster doses—leading to breakthrough infections. As a result, even vaccinated older adults may not achieve the same level of protection against infection with these newer subvariants.

However, our study found that sex, body mass index (BMI), comorbidities, and booster status were not significantly associated with the positivity of any Omicron subvariant (*p* > 0.05). Similar findings have been reported in previous studies, which also concluded that sex and BMI were not significantly associated with Omicron infection^53,54^. Meanwhile, Frank P. Esper et al. conducted a study comparing comorbidity status across different COVID-19 variants, including Alpha, Gamma, and Delta. Their findings showed that patients infected with Omicron who had comorbidities were significantly less likely to be admitted to the hospital, require oxygen therapy, or need ICU admission compared to those infected with earlier variants. Furthermore, a follow-up analysis within the same study revealed that among Omicron-infected patients, there was no significant association between comorbidity and subvariant (BA.1 or BA.2) (*p* = 0.58)^55^. A study by Alexander Wilhelm et al. indicated that minimal neutralization activity was detected after booster administration, with almost no neutralization observed against BA.1 and BA.2 in sera from individuals who had received two doses of any vaccine^56^. Similarly, Ka-Li Zhu et al. found that boosters elicited only limited neutralization activity^57^. Boosters may induce antibodies that bind to the virus but do not effectively neutralize it. Variants of concern, such as Omicron, accumulate mutations in the spike protein and other viral structures, altering the virus’s surface and reducing the effectiveness of antibodies generated by earlier vaccinations or boosters. This results in limited neutralization activity against newer variants. However, despite their limitations, boosters remain important as they provide a broader immune response. The reduced neutralization activity observed in some studies is likely influenced by multiple factors, particularly the emergence of new variants with immune escape properties.

## Strength and limitation

Despite these contributions, several limitations must be considered when interpreting the results. The level of post-vaccination neutralizing antibodies is influenced by numerous clinical, technical, and epidemiological variables, many of which were beyond the scope of this study. Although the study employed a cohort design, neutralization testing for Omicron variants was only conducted at the one-year follow-up. Attrition over the course of follow-ups resulted in a reduced sample size (*n* = 1,117), which may introduce bias or limit generalizability. Additionally, vaccine type distribution across data collection sites varied based on government vaccine supply allocation at the time of rollout. This may have introduced unmeasured site-related confounders. The study also lacked detailed data on the specific type of booster received by each participant, making it impossible to assess differences between homologous and heterologous booster strategies or their respective impacts on antibody responses. These limitations underscore the need for future studies that incorporate more granular data on vaccine regimens, timing, and immunological outcomes. Enhanced data integration would enable more robust assessments of vaccine effectiveness against emerging SARS-CoV-2 variants.

## Conclusion

This study provides valuable insights into the seropositivity of Omicron subvariants (BA.1, BA.2, and BA.4/5) among vaccinated individuals in Malaysia. Our findings indicate that Omicron BA.2 exhibited the highest positivity rate, followed by BA.1 and BA.4/5. Several factors were significantly associated with seropositivity: Chinese and Bumiputera Sabah ethnicity were linked to Omicron BA.1 positivity, while Chinese ethnicity, non-Malaysian status, and vaccination with AstraZeneca or CanSino were significantly associated with Omicron BA.2 positivity. Additionally, Chinese ethnicity and older age were identified as risk factors for Omicron BA.4/5 positivity. Nonetheless, the most effective strategy to reduce the risk of severe infection, hospitalization, and mortality from COVID-19 remains the completion of the primary vaccination series and booster doses. Importantly, this study underscores the need for ongoing surveillance, tailored vaccination strategies, and targeted interventions for at-risk populations, particularly those influenced by demographic and socio-environmental factors. Study conditions and variability such as differences in study design, sample populations, and assay methodologies can influence reported neutralization percentages. Future research and comparative studies should carefully consider these variables to ensure accurate interpretation of results.

## Data Availability

Data is provided within the manuscript and available from the corresponding author on reasonable request.
